# Supplementary material for: Curated and harmonised transcriptomics datasets of interstitial lung diseases
Source: Data Brief. 2025 Oct 14;63:112139. doi: 10.1016/j.dib.2025.112139 (PMC12581653; doi:10.1016/j.dib.2025.112139)
Supplement: Supplementary file 1 [file mmc1.zip › Supplementary_material/RNA-seq/fastQC reports/GSE124685/SRR8397187_fastqc.html]

SRR8397187.fastq.gz FastQC Report 

FastQC Report

Tue 30 Jan 2024  
SRR8397187.fastq.gz

## Summary

- Basic Statistics
- Per base sequence quality
- Per sequence quality scores
- Per base sequence content
- Per sequence GC content
- Per base N content
- Sequence Length Distribution
- Sequence Duplication Levels
- Overrepresented sequences
- Adapter Content

## Basic Statistics

| Measure | Value |
| --- | --- |
| Filename | SRR8397187.fastq.gz |
| File type | Conventional base calls |
| Encoding | Sanger / Illumina 1.9 |
| Total Sequences | 22929158 |
| Sequences flagged as poor quality | 0 |
| Sequence length | 8-366 |
| %GC | 52 |

## Per base sequence quality

## Per sequence quality scores

## Per base sequence content

## Per sequence GC content

## Per base N content

## Sequence Length Distribution

## Sequence Duplication Levels

## Overrepresented sequences

| Sequence | Count | Percentage | Possible Source |
| --- | --- | --- | --- |
| AAGCTCAGGGAGAGCCCTGTTAGGGCCGCCTCTGGCCCTAGTCTCAGACC | 710278 | 3.097706422538499 | No Hit |
| GGGCCACGAGCTGAGTGCGTCCTGTCACTCCACTCCCATGTCCCTTGGGA | 681388 | 2.9717096458579073 | No Hit |
| GCTGCTACAGGAGAATAGCAGACAGGTATAGTTAAGAAACAACTTTATTT | 220860 | 0.963227694623588 | No Hit |
| CTAAGTGCTGTGTTGTCGTTCCCCCTGCTTAAAATAAAGTTGTTTCTTAA | 158744 | 0.6923237216124551 | No Hit |
| GGGAATGTCTACGTGCGTATGCACGTGGCACTCTCTGCCCGAGGTCCGGG | 101891 | 0.44437305547809475 | No Hit |
| GGGGCCACGAGCTGAGTGCGTCCTGTCACTCCACTCCCATGTCCCTTGGG | 93921 | 0.40961382009753694 | No Hit |
| AAAGCCAAGAAAACCAACGATGCCAGATACTGAATTTCTATAGCACTGAC | 86753 | 0.37835231455075674 | No Hit |
| ACACTGAGGACTCTGTTCCTCCCCTTTCCGCCTAGGGGAAAGTCCCCGGA | 83307 | 0.36332341553928843 | No Hit |
| TTTACACCTGCTAGGGTGTTCAAAGGTCAGTGCTATAGAAATTCAGTATC | 79561 | 0.34698613878451184 | No Hit |
| TCTCTGGGCTTCTATTTCGACCGCGATGATGTGGCTCTGGAAGGCGTGAG | 66811 | 0.2913800846939081 | No Hit |
| TGGTTCCTGGCAAGCCCATGTGTGTTGAGAGCTTCTCAGACTATCCACCT | 61074 | 0.2663595409827086 | No Hit |
| ACTTGATCCCAACTCATCTCTCATTTATTTCGGCTTCTTTTATTCCAGGA | 58318 | 0.25433991078084944 | No Hit |
| ATTCTTCCTACCCATGAGCATGGAATGTTCTTCCATTTGTTTGTATCCTC | 51212 | 0.2233488033010196 | No Hit |
| TGACTGGTGATGCTGATATTGGAGTGCATCTGAGGCTGTTGTAGAAATAA | 44975 | 0.19614763001763957 | No Hit |
| AGCGGTTCCGCTGCCCTGAGGCACTCTTCCAGCCTTCCTTCCTGGGCATG | 44162 | 0.192601926333274 | No Hit |
| GGGCCACGAGCTGAGTGCGTCCTGTCACTCCACTCCCCATGTCCCTTGGG | 41362 | 0.18039040072906298 | No Hit |
| ACACCCACCGCAACTGTCTGTCTCATATCACGAACAGCAAAGCGACCCAA | 41154 | 0.17948325882703586 | No Hit |
| CCTCGGCCTCCCAAAGTGCTGGG | 41008 | 0.17884651499195914 | No Hit |
| TCACACTTCATGATGGAGTTGAAGGTAGTTTCGTGGATGCCACAGGACTC | 39407 | 0.17186413910183707 | No Hit |
| GGTTATATCATTGGCAATGTTGTAATCAACAACCCATGTTATTTCTACAA | 37529 | 0.16367369442872695 | No Hit |
| GGGGAATGTCTACGTGCGTATGCACGTGGCACTCTCTGCCCGAGGTCCGG | 34221 | 0.1492466491791805 | No Hit |
| AGTGTGGCGATTCCTCAGGGATCTAGAACTAGAAATACCATTTGACCCAG | 31664 | 0.13809490954704923 | No Hit |
| GGTTATAATCATTGGCAATGTTGTAATCAACAACCCATGTTATTTCTACA | 31631 | 0.13795098799528532 | No Hit |
| AGAGGCCACAGTAAGGCCCATCCGAGGTCCTGAGGAGATCTTTTCTCTCT | 31443 | 0.1371310712761454 | No Hit |
| AGAACGGCGACCGTCAAGGAAAACTGTCACTCTGGGCTCCTTTTTGACCACAGCAGCTATGCGGAAGCAGCTGC | 29767 | 0.12982160095019626 | No Hit |
| AAGCTCAGGGAGAGCCCTGTTAGGGGCCGCCTCTGGCCCTAGTCTCAGAC | 28444 | 0.12405165510220656 | No Hit |
| CAGCATGATTTATAGTCCTTTGGGTATATACCAGTAATGGGATGGCTGGG | 27430 | 0.1196293383298244 | No Hit |
| CACTTGGCTTCACCCGTGTGACTTTCGTAACGGGGAGAGAGAGAAAAGAT | 27090 | 0.11814651022074164 | No Hit |
| AAAAGTGATGGACTGCCCATTGCTGGAGAAGACCTTCTCTCCTACTGTCA | 27061 | 0.11802003370555517 | No Hit |
| CCACCTTCTCCATCGGCTCCACTGGCCTCGTGGTGTATGACTACCAGCAG | 26621 | 0.1161010796820363 | No Hit |
| AAGACCAACGTCAAGGCCGCCTGGGGTAAGGTCGGCGCGCACGCTGGCGA | 26383 | 0.11506310000567836 | No Hit |
| CTTCCGCATAGCTGCTGTGGTCAAAAAGGAGCCCAGAGTGACAGTTTTCC | 25732 | 0.11222392030269929 | No Hit |
| CCGCCTCCGCCGCAGACGCCGCCGCGATGCGCTACGTCGCCTCCTACCTG | 24175 | 0.1054334398149291 | No Hit |
| TGAATCTTGAACTGAGTTCCACTTGTAAACTTCTTGTTTCTTGTGGTTCC | 23312 | 0.10166967317334548 | No Hit |

## Adapter Content

Produced by FastQC (version 0.11.7)
